# Activating and Relaxing Music Entrains the Speed of Beat Synchronized Walking

**DOI:** 10.1371/journal.pone.0067932

**Published:** 2013-07-10

**Authors:** Marc Leman, Dirk Moelants, Matthias Varewyck, Frederik Styns, Leon van Noorden, Jean-Pierre Martens

**Affiliations:** 1 Institute for Psychoacoustics and Electronic Music, Department of Musicology, Ghent University, Ghent, Belgium; 2 Department of Electronics and Information Systems, Ghent University, Ghent, Belgium; UNLV, United States of America

## Abstract

Inspired by a theory of embodied music cognition, we investigate whether music can entrain the speed of beat synchronized walking. If human walking is in synchrony with the beat and all musical stimuli have the same duration and the same tempo, then differences in walking speed can only be the result of music-induced differences in stride length, thus reflecting the vigor or physical strength of the movement. Participants walked in an open field in synchrony with the beat of 52 different musical stimuli all having a tempo of 130 beats per minute and a meter of 4 beats. The walking speed was measured as the walked distance during a time interval of 30 seconds. The results reveal that some music is ‘activating’ in the sense that it increases the speed, and some music is ‘relaxing’ in the sense that it decreases the speed, compared to the spontaneous walked speed in response to metronome stimuli. Participants are consistent in their observation of qualitative differences between the relaxing and activating musical stimuli. Using regression analysis, it was possible to set up a predictive model using only four sonic features that explain 60% of the variance. The sonic features capture variation in loudness and pitch patterns at periods of three, four and six beats, suggesting that expressive patterns in music are responsible for the effect. The mechanism may be attributed to an attentional shift, a subliminal audio-motor entrainment mechanism, or an arousal effect, but further study is needed to figure this out. Overall, the study supports the hypothesis that recurrent patterns of fluctuation affecting the binary meter strength of the music may entrain the vigor of the movement. The study opens up new perspectives for understanding the relationship between entrainment and expressiveness, with the possibility to develop applications that can be used in domains such as sports and physical rehabilitation.

## Introduction

In a study that addressed the effect of music on beat synchronized walking in an open field Styns et al. [1] observed that participants walked faster on music than on metronome ticks. This result suggests that music may affect the human motor system by giving it a boost so that participants take bigger steps than when they walk in synchrony with metronome ticks. When two stimuli have the same tempo and the walking is synchronized with the beat, then it is the stride length that determines possible differences in speed. However, little is known about the acoustical and musical features that cause this effect. Nor is it known whether musical stimuli exist that have a relaxing effect on the participants resulting in them taking smaller steps and thus walking slower than on metronome stimuli.

Inspired by a theory of embodied music cognition [2], our hypothesis is that music may entrain the vigor or physical strength of a movement response. This implies that the flow of a movement may embody expressive aspects of the music on top of a time-entrainment [3]. Studies of time-entrainment addressing diverse aspects of synchronization (see [4] [5] for reviews) including dancing (e.g. [6]–[8]) show that the tight and complex coupling of perception and movement is guided by temporal recurrent patterns in the acoustical structure of the music. These patterns may also influence the cognitive grouping of information into larger musical units at different hierarchical levels [9] [10]. However, certain features of the music (such as the sound pressure level of the bass drum) may also lead to more intense spontaneous hip movements and a higher degree of time-entrainment [11], which suggests an entrainment of the vigor of the movement response in addition to the entrainment of the timing of the movement response. Applied to beat synchronized walking, we assumed that the vigor of the movement response could be most easily observed in the walking speed (or forward stride length). Note that effects on vertical and or sideward movement of the body cannot be excluded but we do not consider these aspects in the present study. The boost effect observed by Styns et al. [1] can be seen as an expression of movement vigor. However, we believe that the set of musical stimuli used by Styns et al. had an activating character. We assume that if some music may activate the beat synchronized movement in such a way that the walking speed increases, it may also work in the opposite way in the sense that some music may relax the beat synchronized movement in such a way that the walking speed decreases. We thus make a distinction between an ‘activating’ effect and a ‘relaxing’ effect and we relate both effects to metronome stimuli that we assume to be neutral because metronome stimuli do not contain sonic energy between the pulses that mark the beat tempo. We reasoned that a lack of such energy cannot possibly entrain the vigor of the movement in an exogenous way. In other words, we assume that an activating and relaxing effect of music on beat synchronized walking is determined by the flow of the sonic energy in the music.

To test our hypothesis we focus on stimuli with a tempo of 130 beats per minute (BPM). This tempo is slightly above the resonance frequency in human movement [12] [13], which is on average 117 BPM [14]. Styns et al. [1] found that an increase of the tempo from 50 BPM to 110 BPM implies an increase in beat synchronized walking speed because more steps with larger stride length are taken. In the range from 130 BPM to 200 BPM, the stride length no longer increases and the walking speed does not increase any more, despite the increase in number of steps taken. The range from 110 BPM to 130 BPM marks a resonance zone where beat synchronized walking is optimal. Within that range the difference in speed in response to music and metronome ticks is most prominent and shows a peak at 130 BPM.

However, we do not know which music has an activating effect and which music has a relaxing effect, nor do we know why it has this effect. Therefore, we start from different musical styles and select music that may have an effect on walking speed. Then we measure the effects and try to identify which qualitative perceptual features and which objective sonic features may be responsible for the observed effects. Accordingly, we conduct a behavioral experiment in two parts. In the first part, participants are asked to walk in synchrony with the musical beat. In the second part, they are asked to listen to the pieces of music and to rate them using a series of bipolar adjectives. To identify the sonic features, we use feature extraction and regression analysis algorithms.

## Methods

### Ethics Statement

The participants signed a consent declaration in which they declare that they are freely participating in the experiment, that they are informed in advance about the task, the procedure and the technology used for measurement. They had the opportunity to ask questions and agreed that recordings of their actions were made. They agreed that recorded data would be used for scientific and educational purposes only. In agreement with the general standards at our university and our faculty, security was guaranteed (our indoor task is not dangerous), and privacy is respected. According to the Belgian law for experiments aiming towards research performed in view of the development of biological or medical knowledge (cf. 7 May 2004 Law concerning experiments on the human person (Ch.II, Art.2, Par.11)), our research is exempt from needing ethical approval as this study only involves behavioral knowledge.

### Participants

The participants were 18 healthy, normally built adults: 7 male, 11 female, aged 22 to 51 (M: 28), between 162 and 187 cm (M: 171) tall and with a weight between 52 and 100 kg (M: 64). Most of them had experience with playing music (14 reported that they play a musical instrument) and 17 of the participants agreed with the statement “I often move to music”.

### Stimuli

The walking experiment was based on 52 musical excerpts and 6 identical metronome sequences. All excerpts and sequences had a duration of 30 seconds and a tempo of 130 BPM (64 beats). Amplitudes were normalized and subjectively checked to minimize the differences in loudness and a short fade-in of 50 ms and a fade-out of 100 ms was applied to each musical excerpt, using CoolEdit. The metronome sequences were generated with Analog Box (http://code.google.com/p/analog-box/). In order to maximize musical diversity, a group of three musicologists collected a set of musical pieces with a tempo of 130 BPM from a variety of different styles. From this set the selection of 52 excerpts was made based on a series of criteria: the three experts should agree that the tempo was 130 BPM, the tempo should be stable throughout the excerpt and the music should have a homogeneous character. Moreover, in the selection process, a preference was given to musical pieces that would probably be unknown to most participants, in order to avoid effects of familiarity as much as possible. Table 1 provides a list of all the musical excerpts that were used. Based on the 52 excerpts and 6 metronome sequences, three playlists (I, II, III) of 58 stimuli (musical excerpts and metronome sequences) were generated by randomly changing the order in which the musical excerpts were presented. The 6 metronome sequences were presented at fixed positions in each playlist, namely, at positions 1, 12, 23, 34, 45, and 58. Between each stimulus, a 5 second break was inserted. Each participant listened to one of the three playlists and all playlists occurred an equal number of times during the experiment. For the second part of the experiment only the 52 musical excerpts were used.

**Table 1:** List of musical pieces.

Id	Composer Performer	Song	Activating Relaxing
1	Mr. de Sainte-Colombe	Courante	r1
2	Hector Zazou	Eye Spy	r2
3	S.E.S.	Sad Song	r3
4	Manu Chao	Minha galera	r4
5	Willem Vermandere	Schoorbakkebrug	r5
6	Penguin Caf Orchestra	Paul’s Dance	r6
7	Al Dexter	Guitar Polka	r7
8	Ken Boothe	Archibella	r8
9	Joseph Haydn	Simphonietta	r9
10	Django Reinhardt	It don’t mean a thing	r10
11	Tokyo’s Coolest Combo	Comment te dire adieu	
12	CPEX	Pinocchio	
13	Bruce Channel	Hey Baby (Dirty Dancing)	
14	Will Tura	Hopeloos	
15	Antonello Paliotti	Sotto e’ncoppa	
16	Rosalie Allen	I wanna be a cowboy sweetheart	
17	Moving Hearts	Hiroshima Nagasaki Russian Roulette	
18	Amuka	Appreciate me	
19	France Gall	Laisse tomber les filles	
20	Nathalie McMaster	Capers jigs	
21	Santana	Primavera	
22	Charles Dieupart	Concerto in a-minor, allegro	
23	Joseph Bodin de Boismortier	“Don Quichotte chez la Duchesse”, Tambourin I	
24	Antonio Vivaldi	Cello Sonata in a-minor, allegro	
25	Georg Friedrich Hndel	Allegro Trio Sonata in g-minor, allegro	
26	traditional Irish	Fred’s tune	
27	anonymous	la Rotta	
28	Suksinder Shinda	Punjabain	
29	Elysium	Interpretation of Dreams	
30	Banda 11 de Enero	Feria de Manizales	
31	Pea Suazo y su Banda Gorda	Aqui, pero alla	
32	Jovanotti	Tutto l’Amore Che Ho	
33	Date of Birth	Aim at El Paso	
34	O-zone	Dragosta din tei	
35	Communards	Don’t leave me this way	
36	Boredoms Jungle	Taitei	
37	Van Halen	Dance the night away	
38	Vasmolon	Lard Ki Labam	
39	Kieran Fahy	McHugh’s	
40	Kosheen	Catch	
41	Jefferson Airplane	Somebody to love	
42	Matthew Dekay	If I could fly	
43	Aqua	Barbie Girl	a10
44	tatu	Not gonna get us	a9
45	Traffic Signs	The big fake	a8
46	Le grand rouge	Parlens d’aimer	a7
47	Junior Jack	The hype	a6
48	Peter Katafalk	Down and Out	a5
49	Kujay Dada	Young Hearts	a4
50	Franceso Veracini	Ouverture no.5 (b-major), allegro	a3
51	Clawfinger	Out to get me	a2
52	Falik	The ballad of El Efe	a1

The first column is the number, the second column specifies the composer or performer, the third column the title of the piece, and the fourth column indicates whether the piece has a relaxing or activation effect. The stimuli are ordered from most relaxing to most activating.

### Procedure

#### Synchronous walking

The walking experiment took place in a sports hall. In the middle of the hall, a circle with a diameter of 15 m was drawn, which served as the pathway for the walking participants. For a video example and sounds, see (http://www.ipem.ugent.be/ActivatingRelaxingMusic). Upon arrival, the participants were briefly informed about the procedure and the goal of the research. Next, they were equipped with a wireless sensor system, called Xbus Kit (http://xsens.com/en/products/human__motion/xbus_kit.php). This kit consists of five MTx sensors that measure acceleration, angular acceleration and the earth magnetic field, each in 3D. These MTx sensors were attached to the top of the right foot, the side of the right ankle, the right knee, the right hip and the right hand, which gives us a detailed image of the participants movements while walking. The MTx sensors were connected to the Xbus Master, which collects the data and sends them to a computer (laptop ACER Aspire 1500) via a Bluetooth connection with a sampling frequency of 50 Hz. Next to the sensor system, each participant received an IPod Nano and a pair of headphones (Sennheiser HD 215). Before starting the experiment, the participants were explicitly instructed to walk in synchrony with the music, sticking to the tempo of the metronome stimulus that they could hear at the beginning of the soundtrack. The walking path was indicated by a circle on the ground. Participants were instructed to walk along the circle whenever they heard a sound through their headphones and to stop when the music stopped. Before they started they heard some music which they could use to adjust the volume. Everybody was asked to choose a comfortable listening level and once this was fixed the volume could not be changed anymore. The excerpts were presented in two blocks of 29 pieces (taking 16 minutes 55 seconds to finish one block). Between blocks, a five-minute break was given, during which some refreshments were offered.

#### Qualitative rating

One or two days after the walking experiment, the participants came to the laboratory to complete the rating experiment. They listened to the same music and rated the excerpts, using nine pairs of bipolar adjectives based on the following [15]: good-bad, happy-sad, tender-aggressive, soft-loud, slow-fast, moving-static, stuttering-flowing, easy-difficult (to synchronize with) and known-unknown. The interpretation of the bipolar adjectives was explained to the participants by means of a short text, before starting the experiment. The adjectives were presented on sheets (one sheet for each musical piece), and were divided by a 10 cm horizontal line. The line was used as a Likert scale, allowing the participant to make a quasi-continuous judgment by putting a mark somewhere along the line. This rating will allow us to clarify the personal motivation and provide a first level of explanation of how the effect of sonic parameters is interpreted by the listener. After finishing the whole experiment, the participants received a voucher of 15 Euro, which they could spend at a well-known multi-media market.

### Feature Extraction and Data-analysis

#### Walking speed

The speed of walking was derived from the outputs of the sensor that was attached to the hip. This sensor information provided the angle with respect to the magnetic north pole. Participants had to walk in a circle, so that for each stimulus the distance between starting position and end position could be calculated using formula 1,
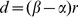
(1)

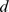
 stands for the distance, 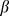 is the angle (in radians) of the end position with respect to the magnetic north pole, 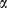 is the angle (in radians) of the starting position with respect to the magnetic north pole, and 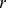 is the radius (here 7.5 m). The speed of walking was calculated by dividing the distance by the duration of the stimulus, which is 30 seconds.

#### Walking tempo

The walking tempo was calculated using acceleration information of the sensor attached to the foot. The tempo was found by taking the peak of a Discrete Fourier Transform (DFT) that was applied to the acceleration data. The size of the DFT was chosen in such a way that the resolution of the DFT bins was equal to 0.5 BPM. As the tempo of only one foot was measured, it was necessary to multiply the obtained DFT bins by 2, so that the value corresponds with half the walking tempo of one foot.

#### Normalizing and averaging the walking speed

In order to assess the effect of the sonic features on walking speed, each song 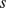 is assigned a unique walking speed 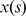. The assignment process is solely based on acceptable trials, defined as trials in which the participant walks in synchrony with the tempo of the stimulus (either a song or a metronome tick). Since the mean walking speed of a participant is bound to depend on his/her physical characteristics, such as length and weight, one needs to assure that the computed 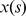 is not affected by these characteristics. Therefore, in a first step, the speed values of the acceptable song trials of participant 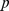 are divided by the mean speed of that participant over the acceptable metronome trials. The underlying assumption is that metronome sequences are neutral in terms of activation and relaxation. Once this normalization is performed, the envisaged 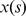 for a song 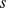 is the mean normalized walking speed over the acceptable trials of all participants for this song. Using this procedure, the walking speed to metronome ticks equals 100 units. Slower and faster walking speeds for songs are rated below or above this value.

#### Extraction of sonic features

It is anticipated that walking speed in synchronous walking is especially affected by the temporal patterns in the music. Therefore, for each of the 52 musical excerpts, a set of 190 sonic features is computed: 2 features are provided by a beat tracker and 188 features emerge from a dedicated feature extractor encompassing three levels of analysis [16], called the frame-level, the beat-level, and the song-level.

Generally speaking, the sonic feature extraction is achieved in three stages. The audio signal is first converted into a stream of acoustic parameter vectors (one vector every 5 or 10 ms; the components of the vector cover subsequent frequency bands). This feature stream is then analyzed per inter-beat interval (IBI) and gives rise to beat-level feature vectors (one vector per beat). In the third stage the time evolution of that beat-level feature in the course of the song, called the feature pattern, is considered as a ‘signal’ and its spectrum is computed at four frequencies, namely 1/2, 1/3, 1/4 and 1/6 of the beat rate. In total, 45 beat-level features were considered, giving rise to 4×47 = 188 sonic features. Each acoustic parameter vector consists of (a) 6 loudnesses (loudness = energy to the power of 0.25) evoked by the outputs of a 6-channel filter bank and (b) 52 evidences for 52 frequencies between 0.1 and 2 kHz and coinciding with the notes on a Western scale. A more technical description is given in the next paragraphs; for more details, see [16].

#### Frame-level analysis

The frame-level analysis consists of two components. The first component considers subsequent fixed length frames of 30 ms long, shifted over 5 ms. Per frame (a time interval of 5 ms), this analysis produces the loudnesses measured in six frequency bands. This is achieved by decomposing the signal into six subband signals by means of six triangular filters with center frequencies of approximately 118, 298, 570, 983, 1609 and 2559 Hz and by measuring the energies of these signals in an interval of 30 ms. The second component considers subsequent frames of 150 ms long, shifted over 20 ms. This analysis produces frame per frame evidences for 52 note-related frequencies ranging from 0.1 to 2 kHz. We consider the note frequencies on an equally tempered western scale.

#### Beat-level analysis

The frame-level features are further considered per beat period. Each beat period is presumed to start with an energetic occurrence that marks the beat at a particular time instance. The beat-level analysis produces 47 sonic features per beat: (i) There are 7 beat-onset features, which describe the total loudness growth as well as the loudness growths in the six subbands at the beat onset time. (ii) There are 3 beat event features, which describe the exact position of the beat onset, the length of the beat event and the skewness of this event. (iii) There are 21 beat period features, which describe the seven frame-level loudnesses (the total loudness and the six subband loudnesses) observed in the course of the beat period following the beat event. Per loudness we first of all retain the mean and the standard deviation of the loudness samples, but we also consider the temporal evolution of the loudness samples in the beat period and we retain the center of gravity of this temporal pattern. (iv) There are 10 beat period features summarizing the information that is retrieved from the pitch saliences computed by the frame-level analysis. The first feature represents the position of the onset of the most salient note found in the beat period. The nine others describe the frequency (in Hz), the pitch class (chroma) and the salience of the three most salient notes. If only two notes are found, the third note is marked by a zero frequency and salience. (v) Finally, we compute 6 beat similarity features describing cosine similarities between subsets of the formerly derived beat onset and beat period features of each two subsequent beat onsets/periods. The considered feature subsets are: (1) the loudness growths in the six subbands at the beat onset, (2) the means of the six subband loudnesses in the beat period, (3) the standard deviations of the six subband loudnesses in the beat period, (4) the centers of gravity of the six subband loudness patterns, (5) the three most salient note frequencies found in the beat period, and (6) the same frequencies after mapping to the chromatic scale.

#### Song-level analysis

In the song-level analysis stage, we consider the beat-per-beat values of each individual beat-level feature as the samples of a ‘signal’ that was sampled at the beat rate. The aim of this analysis is to discover evidences for periodicities of lengths 2, 3, 4 or 6 in such a signal, and to consider these evidences as sonic features. The computed evidences for a particular signal are just the values of the amplitude spectrum of that signal at frequencies of one half, one third, one fourth and one sixth of the beat rate. This implies that every beat-level feature gives rise to four evidences, yielding 188 song-level features in total. These features, together with the outputs of two oscillators residing in the beat tracker, namely, the oscillators tuned to twice or three times the beat rate, complete the set of 190 sonic features characterizing the song.

#### Regression analysis

The goal of the regression analysis is to identify the sonic features that mostly affect the mean normalized walking speed of a song and to obtain an unbiased estimate of the performance one can obtain with these features. If there were enough songs available, one would first divide them in a development set and a test set, and subsequently sub-divide the development set in a training set and a validation set. To select the best sonic features, one would then examine many potentially interesting sonic feature subsets, develop a regression model for each subset in the training set, validate that model on the validation set and finally retain the model with the lowest validation error. To get a performance measure, one would simply evaluate the retained model on the test set.

However, given the low number of songs being available, the procedure mentioned above is not an option and 10-fold cross-validation is used instead. To that end, the 52 songs are divided into 10 folds (8 folds of 5 and 2 folds of 6 songs) and 10 trials are being conducted, one for each fold. In each trial, the considered fold acts as a test set, and the remaining folds as a development set. The feature selection stage of a trial then delivers a set of selected sonic features and speed predictions that are made with these features for the songs in the test fold. After 10 trials, we have speed predictions for all 52 songs and we know how many and which sonic feature are selected in each trial. The root mean squared error (RMSE) between the predicted speeds and the measured speeds of all 52 songs is used as a quality measure for how well the sonic features can predict the walking speeds.

In order to restrict the number of feature combinations to explore in the feature selection stage of a trial, we introduce a feature pre-selection step. Its aim is to identify the 10 potentially most interesting sonic features. We choose 10 because we expect that models using more than 5 features will not generalize well on new songs, so 10 features to select from should be enough. The simplest way to accomplish the pre-selection would be to retain the 10 features which correlate most with the speed measurements. However, this procedure holds a risk of missing a feature that is not very strong on its own but efficient in combination with another feature. Therefore, we tested all 190×189/2 = 17955 possible pairs of two features, we created a regression model per pair and measured the correlation between the predictions of that model and the measurements. We then selected the 10 features occurring most frequently in the 10% best pairs. We actually tried both approaches and the emerging feature sets had 8 features in common. Thanks to the feature pre-selection, it is feasible for the feature selection stage of a trial to examine all combinations of 1 to 10 pre-selected features and to select the best combination. To find the best combination, 9-fold cross-validation is applied to the 9 development folds, as suggested in [17]. The predictions of the walking speeds on the test fold are then obtained by means of a regression model using the combination that is trained on the complete development set. If one does not only want a performance measure, but also a regression model for predicting the walking speeds of future songs, one can select the K features that were selected most frequently in the 10 trials and train a regression model using these features on the all 52 songs. A good value of K would be the maximum number of features that was ever selected in one trial.

## Results

### Synchronization

The criterion that must be met for walking in synchrony with the music is that the number of steps is equal to the number of beats in the music. In 88.7% of the trials, participants synchronized with the musical tempo, which means that most participants had a tempo of 130 steps per minute (SPM). In 9.2% of all trials, participants did not adopt a tempo of 130 SPM, neither did they adopt a multiple or a division of this tempo. This number does not indicate that certain songs were more difficult to synchronize with, but rather that some subjects had difficulties in synchronizing with a large number of songs. In a few cases subjects walked at the double tempo (0.38%) or half of the tempo (1.72%). Only those trials were further analyzed during which the participant walked in synchrony with the musical tempo.

### Effects of Music on Walking Speed

**Figure 1:**
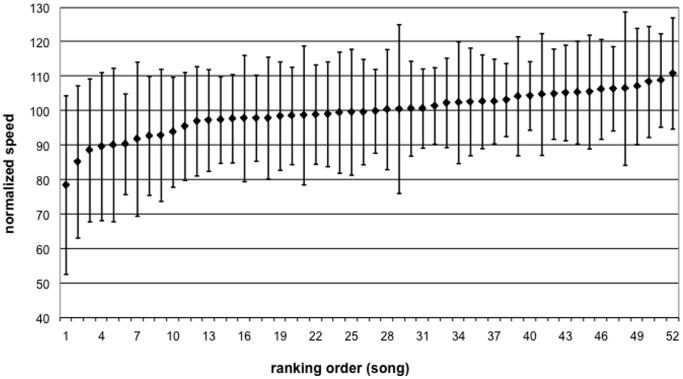
Mean and standard deviation of the normalized walking speed for each song, ranked according to the mean walking speed for the songs 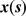.

Figure 1 displays the normalized speeds for the 52 musical excerpts. The figure shows that the speed of walking to music can be slower or faster than the speed measured for metronome ticks. Each participant’s average walking speed with the metronome was scaled to 100%. However, the figure also suggests that there is quite some variability between participants and that the walking speed for quite a number of musical excerpts does not differ from those of metronome ticks. Nevertheless, the figure suggests a significant distinction between the extremes, that is, between low speed or relaxing excerpts (where participants take small steps) and high speed or activating excerpts (where participants take large steps). When we compare the ten fastest (mean = 107.0%) with the ten slowest (mean = 89.4%) and the 10 most neutral songs from the middle (mean = 99.9%), ANOVA shows a significant difference (F(2, 27) = 96.50, p 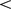.001), with a post-hoc Scheffé test showing highly significant differences between all three the groups. This shows that we can indeed identify music that is activating or relaxing compared to metronome ticks (or neutral music) with the same tempo.

### Regression Analysis

**Figure 2:**
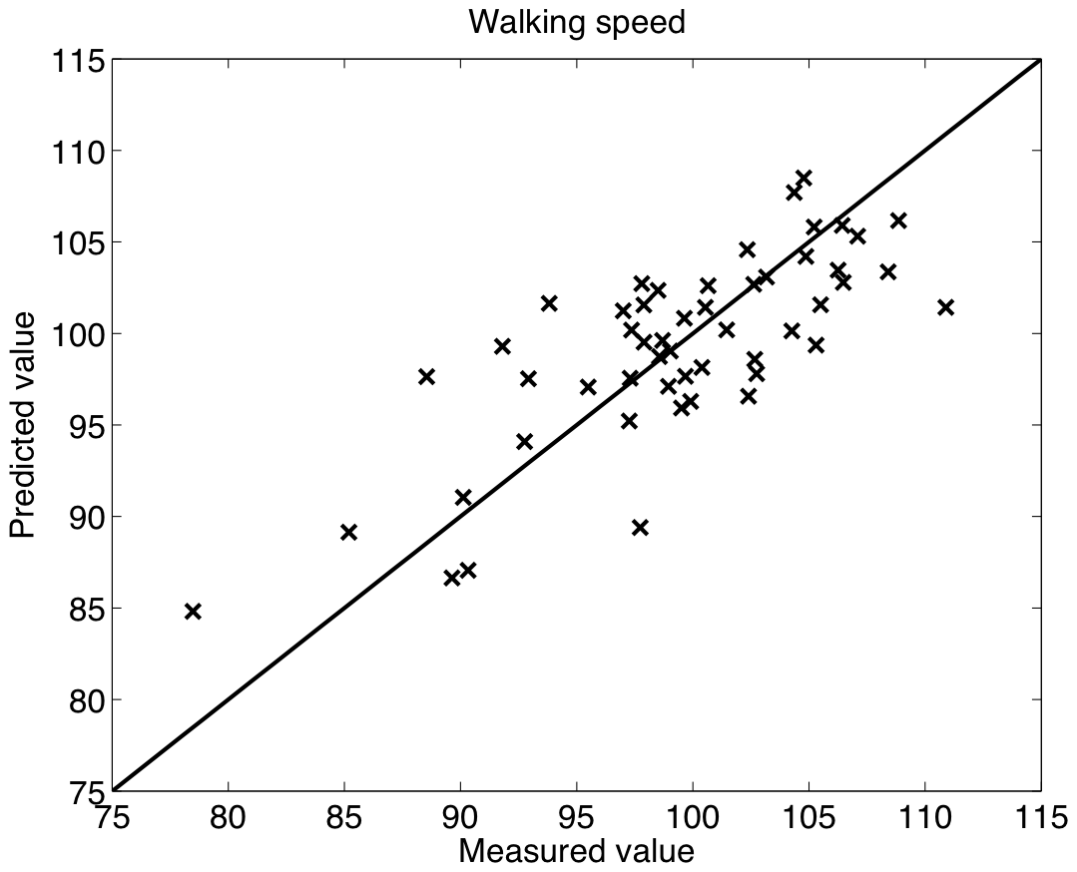
Scatter plot of the speed 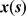 versus their values predicted with a regression model based on sonic features.

A scatter plot of the predicted versus the measured speed values is depicted in Figure 2. This scatter plot shows the predictions of the regression models (one model per test, one test per fold) for the entire dataset. The RMSE between predictions and measurements is 3.98 (recall that the mean value of the measurement is 100). The Pearson Correlation Coefficient (PCC(50)) between the two is 0.77 (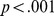), meaning that 60% of the original variance in the measurements is explained by the models (0.60 = square of PCC).

#### Which features are most important

In 8 of the 10 trials, 5 features were used. In the other 2 tests only 4 features were used. The latter 4 features commonly occurred in all 10 tests performed. They are listed in Table 2. One other sonic feature was selected five times, but most others only once or twice. Therefore, it makes sense to focus only on the 4 features that were always selected. Table 2 provides the mean and standard deviation (over 10 trials) of their regression coefficient, as well as their individual PCC with the measurements. These PCCs show that features 178 and 176 exhibit the strongest correlations. Feature 178 alone, can already explain 44% of the variance (PCC = −0.67). Note that five features exhibiting a higher absolute correlation with the measurements than feature 131 were not selected more than 2 times. Apparently, they provide no complementary information. Since feature 131 was always selected and since it was not retained by the more simple pre-selection method mentioned above, a final evaluation with the feature set emerging from that simple pre-selection was conducted as well. This feature set provided a much smaller gain for feature 178 alone: PCC = 0.72, explained variance of 51% and only 2 features (178 and 176) that were selected 10 times.

**Table 2:** The most frequently selected sonic features (out of ten models) for walking speed.

Id	Walking speed	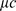	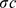	N	PCC
176	Evidence for a period of 6 beats in the similarity between the standard deviationsof the six loudness features in subsequent beat periods	−244	49	10	−0.53
131	Evidence for a period of 4 beats in the salience of the most salient note in abeat period	229	33	10	0.40
152	Evidence for a period of 6 beats in the frequency of the third most salient notein a beat period (frequency = 0 if no third note is present)	−275	42	10	−0.42
178	Evidence for a period of 3 beats in the similarity between the centroids of thesix loudness features in subsequent beat periods	−362	63	10	−0.67

For each feature we list the feature number (Id), the mean (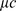) and standard deviation (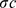) of the regression coefficients for these features in the models and the number of times (N) (0. 10) the feature was selected.

#### What do the features represent

Features 176 and 178 are both derived from an analysis of the individual loudness patterns in subsequent IBI’s (recall that a pattern is defined as an evolution in time). In the case of feature 176, the variances of the six loudness patterns in each IBI are computed, and the cosine similarity between the variance vectors measured in two subsequent IBIs is considered as a feature of the beat between those IBIs. Feature 176 is high if the spectral analysis of the temporal evolution of this feature over the song excerpt reveals the clear presence of a frequency of one sixth of the beat rate. In the case of feature 178, the center of gravity (the centroid) of each loudness pattern in an IBI is computed and the cosine similarity between the centroid vectors measured in two subsequent IBIs is considered as a feature of the beat between those IBIs. Feature 178 is high if the spectral analysis of the temporal evolution of this feature reveals the clear presence of a frequency of one third of the beat rate.

In order to get some idea about the relationship between sonic features and music properties one may consider the example of music in which the loudness patterns in subsequent IBIs differ. Such differences can emerge from small timing deviations of instruments with respect to each other (known as laid-back) or from differences in the swellings of articulated tones (due to a crescendo and decrescendo). Both of these phenomena are known to affect the expressiveness of the music and they cause a disturbance of the dominant binary meter (due to delayed onsets, or the suggestion of syncopation).

Features 131 and 152 are both derived from an analysis of the note evidences measured in subsequent IBIs. In the case of feature 131, the mean evidence of the most salient note in each IBI is considered as a feature of the initial beat of that interval. Feature 131 is high if the spectral analysis of the temporal evolution of this feature reveals the clear presence of a frequency of one fourth of the beat rate. In the case of feature 152, the frequency of the third most salient note in each IBI is considered a feature of the initial beat of that interval. Feature 152 is high if the spectral analysis of the temporal evolution of this feature reveals the clear presence of a frequency of one third of the beat rate. Given the fact that the frequency is set to zero if only one or two notes are found in the IBI, feature 152 is high if three notes are found only once every three IBIs.

Interestingly, the selected features span periodicities of 3, 4 or 6 beats, whereas occurrences of bursts or energy distributions of onset features were not selected. This indicates that the features are indicative of fluctuation and emphasis that covers subsequent beat periods. In addition, the features measure phenomena that cover the entire sound spectrum rather than just a part of that spectrum (e.g. a single subband). Therefore, we can say that the features measure global temporal phenomena (with periods up to 6 beats) and global spectral phenomena (all 6 subbands).

To sum up, the analysis suggests that articulated fluctuations of loudness and emphases of pitch are expressive characteristics that may weaken or strengthen the binary meter. The weakening of the binary meter is due to expressive properties of composition and performance that leave traces of ternary components. When a spectrum is made of the IBI comparisons, the spectral peaks will typically articulate the binary meter due to the fact that the basic structure of the music is predominantly binary. However, when the articulated expression implies deviations from the binary meter (as in laid-back or crescendo-decrescendo notes), these peaks will be broader and spread out to the ternary region. Therefore, the measurements at three or six beats do not necessarily reflect peaks that mark a clearly audible ternary structure of the music. They rather reflect articulated expressive characteristics of the performance that is predominantly subsumed under the binary meter and affect that meter. In contrast, the absence of this type of articulated expression and the presence of other additional binary features emphasize a more strict binary meter.

#### How do the features affect the walking speed

The regression coefficients of features 176 and 178 are negative, which means that they are associated with the desire to take smaller steps. The regression coefficient of feature 131 is positive, which means that it encourages the participants to take bigger steps. A more detailed analysis indicates that most activating songs had a simple tonal pattern in the sense that only one or two salient notes per IBI were found, whereas the most relaxing songs had a more complex tonal structure (many IBIs with three notes were discovered). Apparently, a more complex tonal structure in combination with a weakened binary meter tends to diminish the enthusiasm of the participants to take bigger steps. In general terms we can say that traces of expressive articulation in the ternary meter are characteristic for relaxing music, whereas an absence of these traces and an emphasis on the binary meter is characteristic for the activating music.

### Qualitative Evaluation of the Activating and Relaxing Excerpts

We selected the 10 excerpts which induced the highest average speed and the 10 fragments which induced the lowest average speed in the walking experiment. Excerpts that induce a high speed are labeled activating (marked with 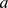 in Table 1, where 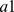 is the most activating excerpt), while excerpts that induce a low speed are considered relaxing (marked 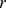 in Table 1, where 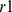 is the most relaxing excerpt). First, we checked whether the sonic features differed significantly between the activating and relaxing excerpts. Then we carried out a qualitative analysis of the style of the excerpts and examined if differences between the two groups also appear in the ratings derived from the second part of the experiment.

To check whether sonic features differ, we took the values of the five sonic features for the sets of 10 activating and 10 relaxing excerpts, and we applied t-tests to see if they differ significantly. This is indeed the case for all four features: feature 176 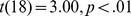; feature 131 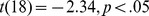; feature 152 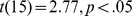; feature 178 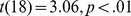. This indicates that these particular sonic features indeed make a difference between the groups of activating and relaxing excerpts.

**Figure 3:**
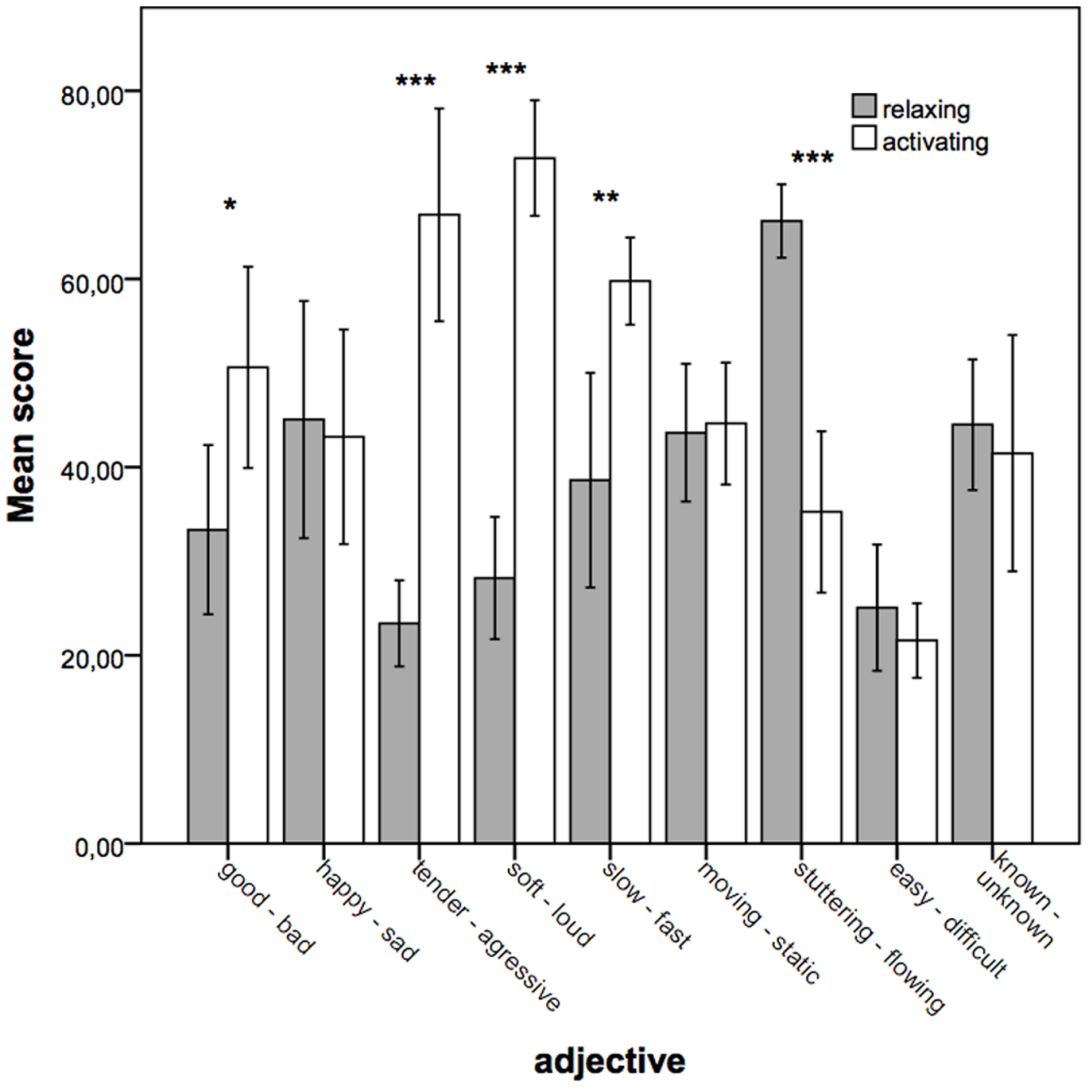
Average score of nine bipolar adjectives (with 0 indicating the first adjective and 100 indicating the second adjective) for the 10 relaxing excerpts (grey bars) and the 10 activating excerpts (white bars). The stars indicate significance levels.

Figure 3 shows relaxing excerpts and activating excerpts in relation to the qualitative descriptors as they were rated by the participants during the second part of the experiment. Mann-Whitney tests on the mean ratings per excerpt showed that the adjectives bad-good, aggressive-tender, loud-soft, fast-slow and fluent-stuttering are ranked significantly different in both groups. In particular, this means that activating is related to the qualitative features bad (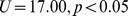), aggressive (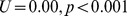), loud (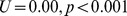), fast (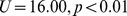), stuttering (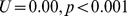). In contrast, the adjectives sad-happy, static-moving, difficult-easy and unknown-known are not ranked significantly different. This analysis suggests that sound qualities related to loudness, timbre, texture are perceptually more important than adjectives that probe subjective experiences related to emotions, difficulty, familiarity, and taste. Overall, participants are consistent in their observation of qualitative differences between the relaxing and activating musical stimuli.

### Style Description of the Activating and Relaxing Excerpts

A stylistic interpretation of sonic features is challenging because the sonic features may capture combined musical configurations. After having listened to the most activating and most relaxing musical pieces, we were left with the following impressions. Activating music tends to exhibit (i) less variation in the musical expression, (ii) a continuous strength that is typically supported by a bass that is active at a subbeat metric level, resulting in a more or less constant loudness over several measures, (iii) a downbeat characteristic (on the first and third beat), with the strongest pitch on the first beat, (iv) short melodic motives using short notes that are often repeated, (v) a lot of noise in the timbre. Relaxing music tends to have (i) more variation in the musical expression, (ii) a variable strength that clearly articulates the played notes, leading to fluctuations in the loudness patterns, (iii) an upbeat characteristic (on the second and fourth beat) with harmonic support on the second and fourth beat, (iv) longer melodic motives and phrases, and legato notes, (v) a more transparent and bright timbre. Obviously, these descriptions somehow summarize a general difference between the most activating and the most relaxing musical excerpts. As far as genre is concerned, pop-techno is more prominent in activating excerpts, while jazz-reggae is more prominent in relaxing excerpts. In general, the relaxing excerpts contain more variance in phrasing and melody, whereas the activating excerpts have a more equalized phrasing stressing a binary structure.

## Discussion

### Musical Interpretation and Theoretical Model

The present study shows that in beat synchronized walking, temporal patterns in music may influence the walking speed (or stride length). This result supports the hypothesis that music may entrain the vigor of the movement response on top of time-entrainment. The findings show that music can influence a control mechanism for movement activation and movement relaxation and that participants are consistent in their observation of qualitative differences between relaxing and activation musical excerpts. The effect can be predicted by a small number of features extracted from recurrent temporal patterns in the sonic energy of the music. However, the musical interpretation of these features is challenging given the fact that the features may capture the combined effect of different musical phenomena. The features suggest that fluctuations in loudness and pitch affect the perceived meter strength. Weakening of the binary meter strength affects the strength of ternary components and this contributes to a relaxing effect. The absence of these fluctuations implies a more regular binary meter which, in combination with a pitch emphasis on binary components, contributes to an activating effect.

There are different explanations for this effect. One possibility is that expressive patterns in a binary meter cause an attention shift [9] to a larger time period (namely ternary rather than purely binary), thus affecting the movement vigor and resulting in a slowing down of the speed while keeping the tempo constant. Similar attentional shifts have been documented in studies that address synchronized finger tapping to music and artificial stimuli [18]–[21]. However, expressive aspects of music are subtle and their effect on movement may also occur at a subliminal level. Thus, rather than speaking of an attention shift, the effect may be due to a direct audio-motor coupling in which articulated fluctuations affect the strength of the binary meter. This is then directly translated in the vigor of the movement response. Nozaradan et al. [22], [23] show that the brain reflects beat and meter in accordance with the resonance frequency of moving [12]. Metric structures thus resonate in the brain and there is evidence that they entrain the motor system [24]–[29]. The connection with imagery forms a further basis for a music-driven perception-action coupling that may influence the vigor of the movement [30], [31]. In addition, it is possible that the continuous strength of the activating music is reflected in a continuous strength of the movement response. Van Dyck et al. [11] provides evidence that the sound pressure level of the bass drum may entrain movement intensity and synchronization. The absence of articulated loudness fluctuations and presence of a continuous activity of the bass at subbeat level gives a particular emphasis to the binary meter and comprises an energetic component that may contribute to the activating effect.

### Qualitative Descriptors

The analysis of sonic features has been complemented by a rating task, showing that the participants perceive differences between activating and relaxing music in terms of the adjectives bad-good, aggressive-tender, loud-soft, fast-slow and fluent-stuttering. However, the adjectives sad-happy, static-moving, difficult-easy and unknown-known do not mark such a difference. This suggests that personal taste is not a motivating factor. One could expect people to walk faster when they listen to their familiar music, to music which generates positive vibes or to music which is easy to synchronize to. However, we detect no difference in perception between activating and relaxing music with regards to adjective pairs such as sad-happy, easy-difficult and unknown-known. Interestingly, the relevant qualitative descriptors seem to stress an experience of sonic energy, as music with an activating effect is associated with the adjectives aggressive, loud, fast, and stuttering. Music with a relaxing effect is associated with the adjectives tender, soft, slow, fluent. It is tempting to associate this type of descriptors with sonic features that characterize global spectral and temporal patterns.

### Music in Sports and Rehabilitation

Although several studies have looked at the effects of music on human movement, only a few studies have examined in detail synchronized walking movements in relation to musical parameters. Studies that addressed the non-synchronized use of music during sports exercise showed for example that music influences motivation, and that the effect is typically determined by sonic features such as tempo and loudness levels [32]–[34]. Studies that looked at the synchronized use of music, such as during treadmill-walking [35] or running [36] showed that an effect on body-related properties such as effort (heart rate, perseverance) occurs. Apart from objective measurements, the experiences of participants have been analyzed using questionnaires that probe effort sensation, perceived exertion, arousal, emotion, and so on. It has even been suggested that positive experiences associated with synchronous body movement are linked to an increase in neuromuscular or metabolic efficiency [37]. Also in physical rehabilitation programmes, music has been used to influence human movement. For example, music has been used to improve gait patterns in stroke patients who have suffered a stroke [38]. In addition, it is known that sonic cueing may enhance gait coordination in Parkinson patients [39], and that rehabilitation patients who walk on music can walk longer than patients who do not walk on music [40], [41]. However, most studies that address the effect of music on walking focus on general musical parameters, such as the difference between music and no music, fast and slow music tempo, or low and high intensity levels. Less attention has been devoted to more fine-grained sonic features. Furthermore, it should be noted that the effect of music on synchronized walking speed in a context of treadmill walking is complicated due to the fact that the treadmill interface imposes a fixed speed, and therefore influences the step size upon synchronized walking.

The results of the present study are relevant for the hypothesis [35] that music, in contrast with no-music, can alter psychomotor arousal and thus can act as a stimulant or sedative. Our study shows that music at 130 BPM can be activating or relaxing, depending on the musical style and structure, which is reflected in features that mark temporal distribution of sonic energy. In addition to [36] who used two musical pieces that were rated as either motivational or non-motivational (oudeterous) music by the participants, and where both musical conditions resulted in higher speeds compared to the no-music condition, our study shows that finer distinctions are possible, and that music of the same tempo (130 BPM) can indeed have an activating or relaxing effect on the speed of synchronous walking. Moreover, our study shows that it is possible to identify sonic features that predict the effect.

Music has a performance- and motivation-increasing effect on joggers, especially on untrained people [42]. Recently, applications have been developed that aim at exploiting music for walking, such as StepMan [43] and Djogger [44]. Up to now, these devices measure the walking tempo and provide music with the same tempo. However, in developing sports applications, where joggers follow a training program based on synchronized walking and running with music, one may question whether tempo is indeed the only criterion that determines the walking speed, and whether additional stylistic sonic features should be taken into account in order to influence aspects of movement vigor.

### Music and Muscular Bonding

The results of our study may contribute to a better understanding of the muscular bonding effect [45]. McNeill observed that throughout history, one of the main functions of beat synchronized walking (such as marching) is to arouse a euphoric fellow feeling within a group of individuals. This feeling is typically achieved through prolonged and rhythmic muscular motions that can be supported by the music, such as during working on the field, or during exercise and marching. The effect is called muscular bonding, and it puts forward the important role of the human body as a mediator between sonic properties of music (tempo, loudness) and experiences (fellow sympathy, arousal). This viewpoint is a core component of the embodied music cognition research paradigm, in which the relationship between human motion and musical experience is seen as a cornerstone of musical signification [2]. It is possible that vigor entrainment can be linked with a fellow feeling effect (see e.g. [46]). However, this effect needs further study and clarification.

### Conclusion

This study concentrates on the effects of beat synchronized walking in human beings. If human walking is in synchrony with the beat and all musical stimuli have the same tempo (namely 130 BPM), then consistent differences in the walking speed of people can only be the result of music-induced differences in stride length. Taking bigger or smaller steps in response to the particular nature of the music can then be directly coupled with the vigor of the movement response. The major contribution of this study is that: (i) It shows that the entrainment of music and movement involves two different components namely, timing and vigor. The entrainment of timing is related to tempo (and phase) of the movement response, while the entrainment of vigor is here defined in terms of activation and relaxation. We believe that this finding sheds new light on the embodiment of music, in particular how music may influence the physical strength of a movement. (ii) The study also shows that the entrainment of the vigor of a movement response to music is due to expressive musical features that are reflected in the musical meter. A limited number of sonic features can predict the difference between activating and relaxing music and hence the difference in vigor response. The sonic features focus on recurrent patterns of fluctuation in the binary and ternary meter strengths. This finding is interesting in that it reveals a relationship between expressiveness and meter. Overall, our study opens new perspectives for understanding the relationship between musical entrainment and expressiveness, with the possibility to develop applications that can be used in domains such as sports and physical rehabilitation.
